# Suture Bridge Technique for Arthroscopic Repair of Posterior Cruciate Ligament Tibial Avulsion

**DOI:** 10.1016/j.eats.2025.103558

**Published:** 2025-04-28

**Authors:** Shengkun Wu, Ruoxi Zhang, Yi Zheng, Yubin Long, YuSheng Li, Xiaoqian Men, Jiangtao Dong

**Affiliations:** a Third Hospital of Hebei Medical University, Shijiazhuang, Hebei, China; b The First Central Hospital of Baoding, Baoding, Hebei, China; c Xiangya Hospital of Central South University, Changsha, Hunan, China

## Abstract

Arthroscopy has gradually become the routine method to treat posterior cruciate ligament tibial insertion avulsion. However, arthroscopic surgery possesses the advantages of being minimally invasive, having a good appearance, and having no need for revision surgery for internal fixation. Compared with open surgery, it is limited by disadvantages such as unstable fixation, unsatisfactory reduction, and fracture fragment displacement. After comparing the various methods proposed in the literature, the "suture bridge" technique, which is commonly used for rotator cuff treatment, is used for repair of posterior cruciate ligament tibial insertion avulsion in combination with the double posterior medial arthroscopic portals, is applied by our team. This surgical technique enables the even distribution of sutures above the fracture fragment, reduces the possibility of suture displacement, and enables excellent reduction under arthroscopy while fully preserving the posterior septum.

The posterior cruciate ligament avulsion (PCLA) is a special type of posterior cruciate ligament (PCL) injury. This injury can lead to varying degrees of knee joint instability and may hinder the ability of the patient to participate in sports. Moreover, it also triggers the development of secondary osteoarthritis.^1^ For Meyers-McKeever III type PCLA (Fig 1), surgery often is required to the reduction of fractured fragment to restore joint stability. The surgical treatment options include open surgery and arthroscopic minimally invasive surgery. With the increasing maturity of arthroscopic technology, an increasing number of surgeons adopt all arthroscopically reduction surgeries. The surgery possesses the advantages of a small incision, and no indication for a revision internal fixation surgery. Moreover, other intra-articular lesions can be concurrently treated.^2^

**Fig 1:**
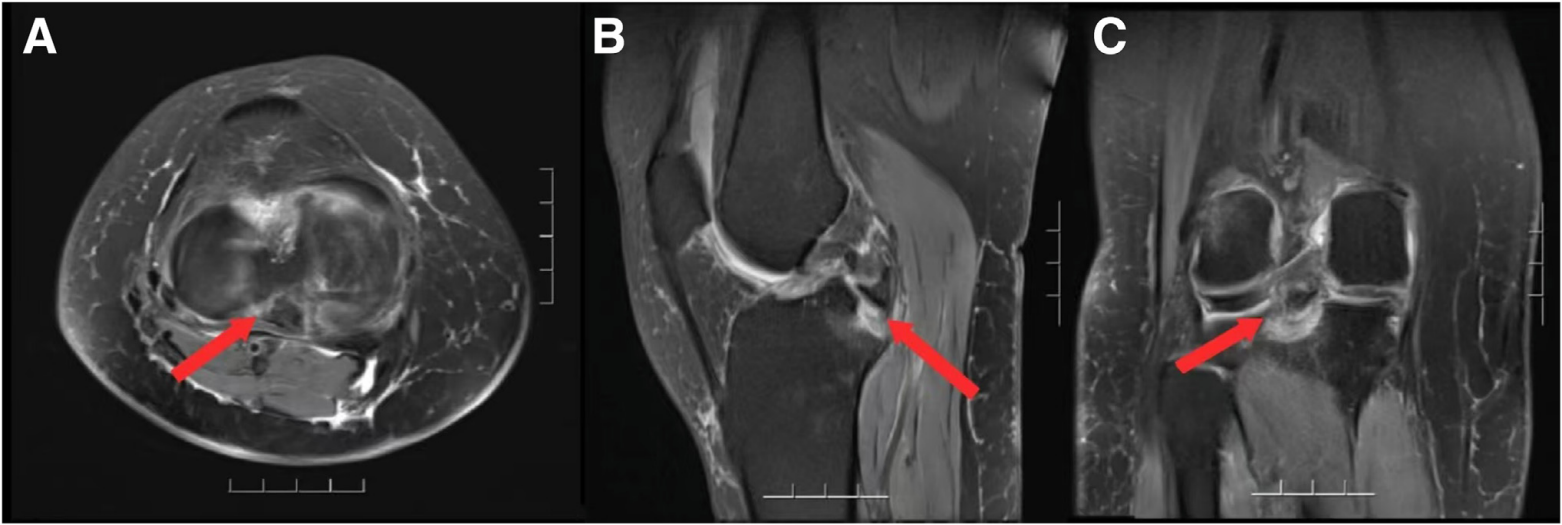
Magnetic resonance imaging scans of the patient before surgery (patient side: right). (A) Transverse plane. (B) Sagittal plane. (C) Coronal plane.

The arthroscopic treatment options depend on the type, size, degree of displacement, comminution, and concomitant injury of the fracture. Various surgical techniques, such as adjustable single- or double-button fixation, suture strapping fixation, hollow lag screw fixation, etc., have been reported in the literature for the treatment of PCLA. However, compared with open surgery, arthroscopic surgery is limited by disadvantages such as unstable fixation, incomplete reduction, and easy displacement of postoperative fracture fragments.^3^^,^^4^

Combining the aforementioned methods and our clinical experience, the rotator cuff repair "suture bridge" technology is applied by our team for the treatment of PCLA. FiberWire (Arthrex, Naples, FL) is used to wrap the PCL-attached bone in the joint space to achieve good tension distribution above the fracture by using the double posterior internal arthroscopic portal. An external row of rivets is used outside the joint to stabilize and affix the bone fragments.

## Surgical Technique

The procedure is carried out with the patient under general anesthesia and placed in a supine position. A knee examination is performed on the knee joint while the patient is under general anesthesia. The 2 tests are compared with the postoperative examination to evaluate the surgery outcome.

**Fig 2:**
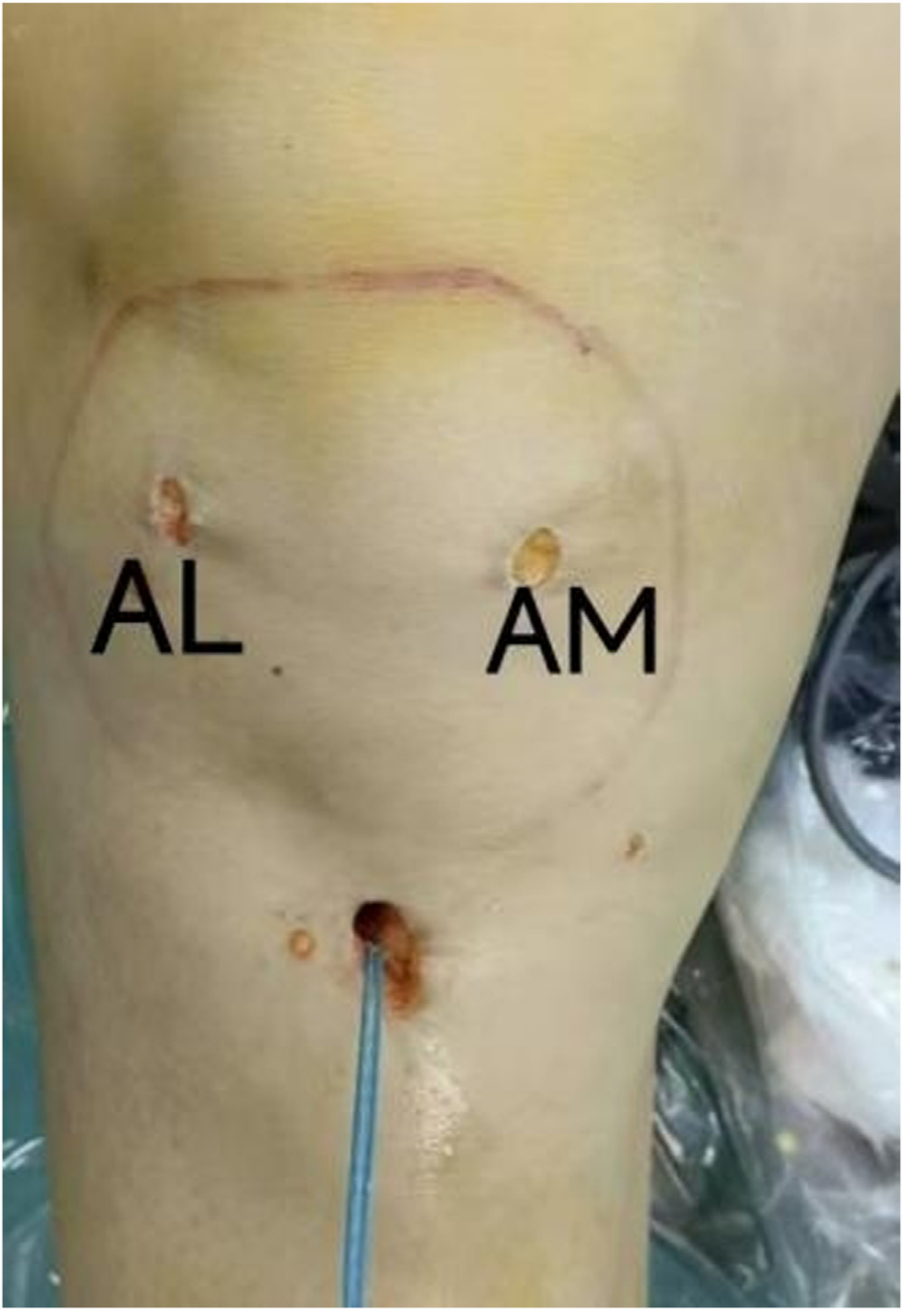
Posterior cruciate ligament avulsion—schematics of surgical techniques. (AL, anterolateral; AM, anteromedial.)

**Fig 3:**
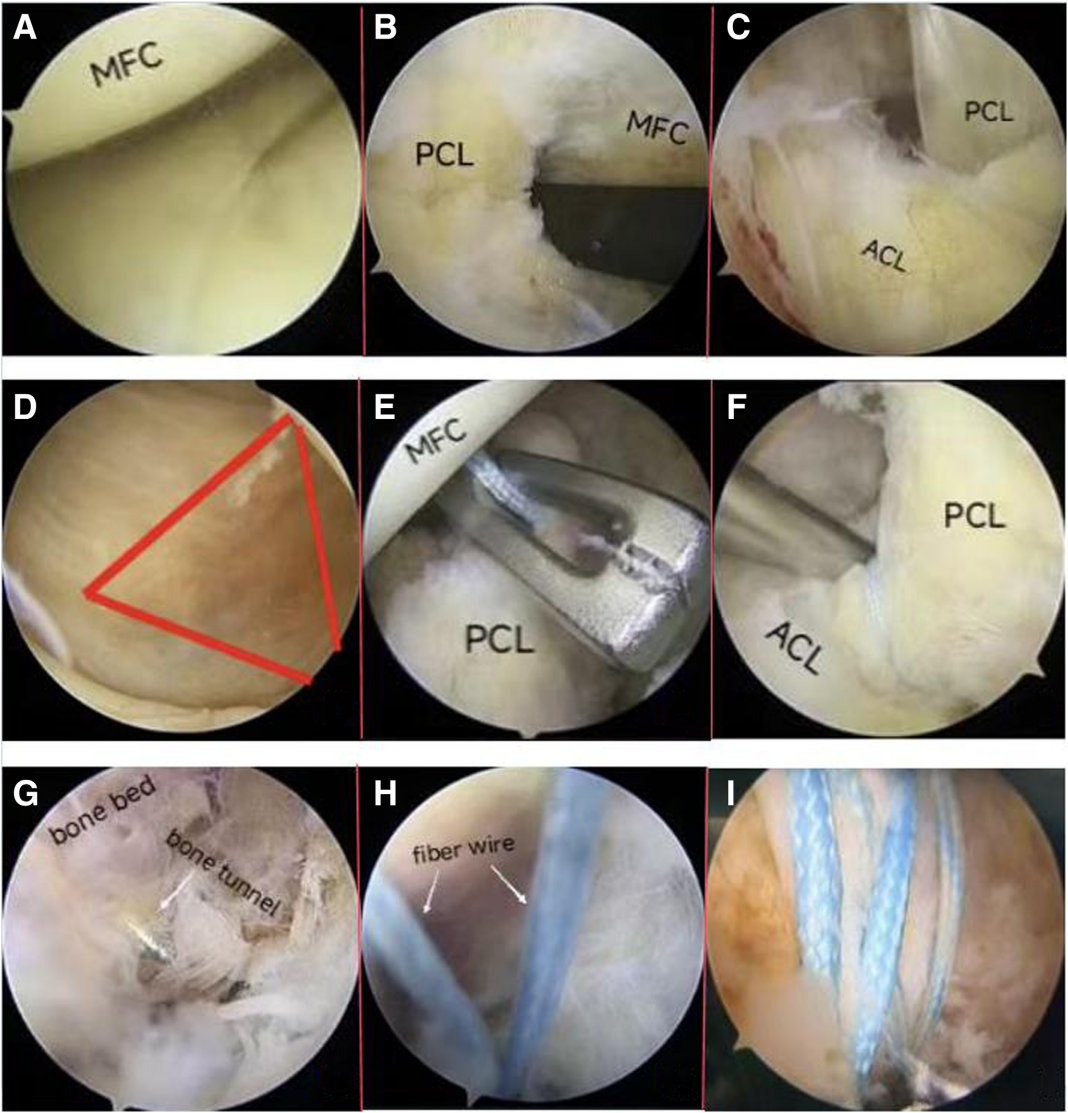
(A) Using a probe to explore the meniscus, the meniscus was found to be stable, and no treatment was performed. Side, right; position, supine; anteromedial. (B) The operative path is created between the ACL and the PCL. Side, right; position “4”; anteromedial. (C) The pathway between the PCL and MFC fossa is created. Side, right; position “4”; anteromedial. (D) The triangle is enclosed by the fold of the medial head of the gastrocnemius muscle. Side, right; position “4”; anteromedial. (E-F) The PCL is bandaged with fiber wire. (E) Side, right; position “4” low posteromedial. (F) Side, right; position “4”; anteromedial. (G) Drill out the bone tunnel with a 2.5-mm guide needle. Side, right; position “4”; low posteromedial. (H) Both ends of the FiberWire and PDS wire ring are pulled out from HPM in joint cavity. Side, right; position “4”; low posteromedial. (I) Suture bridge. Side, right; position “4”; low posteromedial. (ACL, anterior cruciate ligament; MFC, medial femoral condyle; PCL, posterior cruciate ligament; PDS, polydioxanone.)

First, the anteromedial portal and anterolateral (AL) portal are established to perform routine arthroscopy and joint cavity cleaning (Fig 2), including hematoma removal and examine meniscus (Fig 3A, Video 1), anterior cruciate ligament (ACL) and PCL exploration.

### Procedure

**Fig 4:**
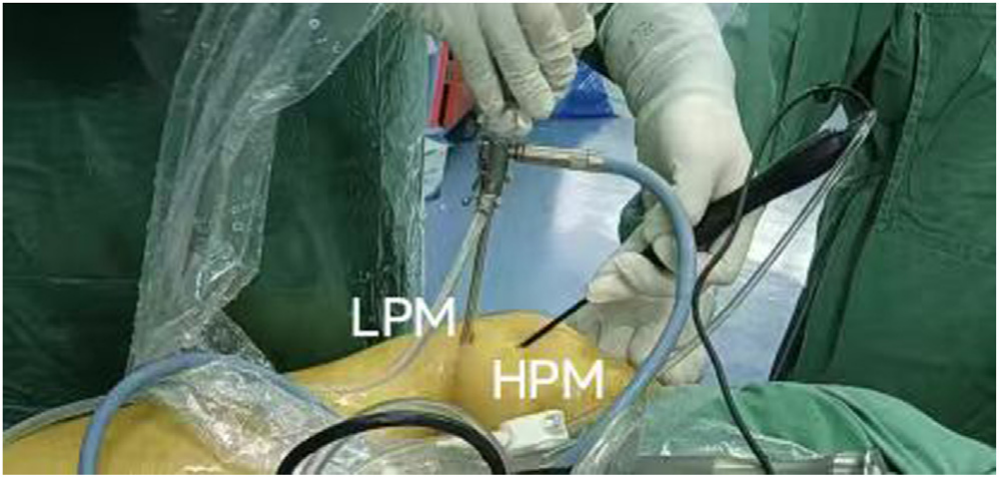
The high posteromedial (HPM) portal is opened 3-4 cm above the low posteromedial (LPM) portal (side, right; position “4”).

Two pathways are established for ease of the procedure: one between the ACL and the PCL, and the other between the PCL and the medial wall of intercondylar notch (Fig 3 B and C). Next, the arthroscope is inserted into the posteromedial compartment through the pathway between PCL and the medial wall of intercondylar notch. The "soft spot" with the most obvious deformation in the ramp region of the meniscus, the posterior condyle of the femur, and the fold of the medial head of gastrocnemius are located (Fig 3D). A scalpel is used to make a skin incision to establish a low posteromedial (LPM) portal. The arthroscope is removed from the AL portal and transferred to the posterior medial compartment through the LPM portal with an exchange rod. A high posteromedial (HPM) portal is opened at a distance of 3 to 4 cm from the LPM under direct vision (Fig 4). The LPM portal serves as the observation portal, and the portal serves as the work portal. In the case of observation through arthroscopy, the tissue between the PCL and the posterior joint capsule is cleaned with an orthopaedic planer to expose the fracture block.

Under arthroscopic observation, use a wire grabber to insert the one end of the first FiberWire into through the pathway between the PCL and the medial wall of intercondylar notch by the AL portal. The other end is inserted through the pathway between the ACL and the PCL. The FiberWire wrap, the PCL, and the 2 ends of the FiberWire are pulled out of the body from the HPM portal (Fig 3 E and F). The PCL reconstruction locator (Smith & Nephew, London, England) is inserted through the anteromedial portal and is positioned to the external posteriorly below the tibial fracture end (2-3 mm below the fracture line). A 2.5-mm guide needle is used to drill out the bone tunnel 1 (Fig 3G). After the completion of the bone tunnel, the PDS is imported into the joint cavity with the dural needle, and both ends of the FiberWire and PDS are pulled out of body from the HPM portal with the wire grabber (Fig 3H). Both ends of the FiberWire insert into the polydioxanone (PDS) ring in vitro and pull out from the bone tunnel 1. The second FiberWire passes through the ACL and PCL pathway, and bone tunnel 2 is established in the medial posterior and lower part of the tibial fracture (Fig 3I). The PDS is inserted into the joint cavity and both ends of the second FiberWire and PDS are pulled out of body from the HPM portal. Both ends of the FiberWire are inserted into the PDS ring in vitro and are pulled out from the bone tunnel 2.

**Fig 5:**
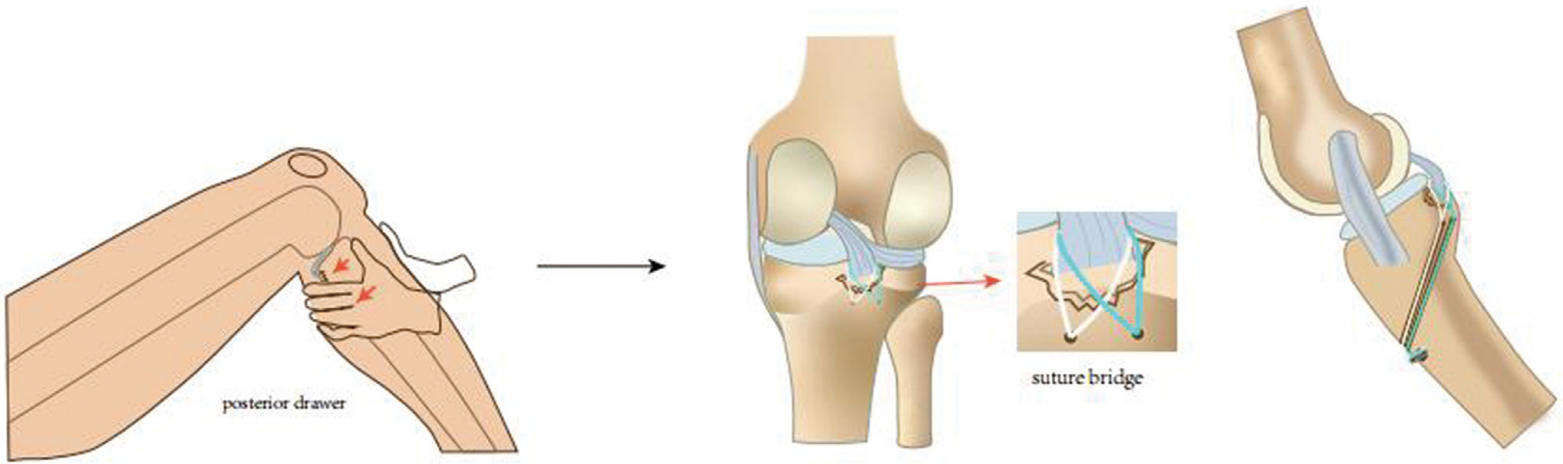
Fixation is performed under the “anterior drawer test”.

After the FiberWires are pulled out through the 2 bone tunnels, a special open-circuit cone of the external row anchor is used to make a hole penetrate over the cortex in the anteromedial aspect of the proximal tibia about 1 cm below the 2 bone tunnels. When the knee joint is in a neutral position at 90°, the PCL tension is restored under the action of the "anterior drawer test" to push the tibia forward (Fig 5). At the same time, the 4 FiberWires are wrapped to the external row anchor and the 4 FiberWires are tightened. The outer row anchor is driven into the hole to fix it.

### Postoperative Rehabilitation

**Fig 6:**
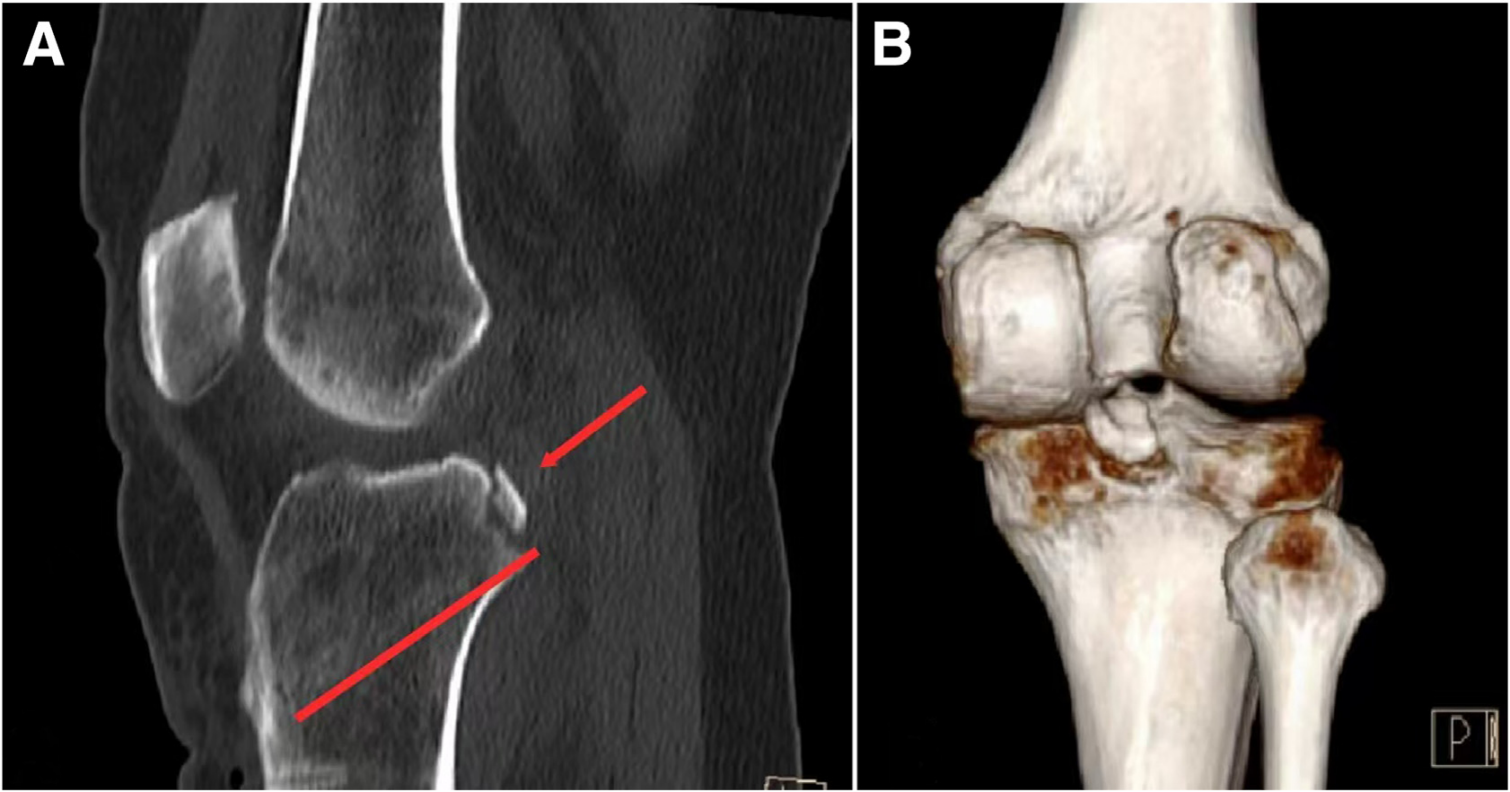
Computed tomography scan and its 3-dimensional reconstruction of the patient after surgery (patient side: right).

After the operation (Fig 6), the patient’s knee joint is immobilized in a knee joint brace with a posterior tibial support to prevent the posterior tibial from sagging. Patients are encouraged to perform active straight leg raises and static quadriceps-strengthening exercises on the first day after surgery. The patient is required to use non−weight-bearing with bilateral axillary crutches for a month.

## Discussion

The LPM and HPM dual posterior internal portal adopted can preserve the posterior septum to maintain a large operating space and a clear surgical field of view in the posterior medial compartment of the knee joint. The physiological anatomy of the posterior septum preserved during surgery is crucial for the postoperative motor function recovery of patients with PCL tibial avulsion. Ramos et al.^5^ found that the posterior septum distributed the arterial blood network in the middle of the knee and contained an abundant number of type II and IV mechanoreceptors. Therefore, preserving the posterior septum during the operation can ensure the supply of blood flow in the posterior septum and effectively reduce the neurosensory disorders caused by the loss of proprioceptor, thereby speeding up the efficiency of rehabilitation.

The ^“^suture bridge” technique has been widely applied in arthroscopic surgery for rotator cuff tears. As a double-row suture technique, the “suture bridge” has been favored by many orthopaedic surgeons because it improves the contact area, increases the pressure load, and shortens the surgical time.^6^ When using the “suture bridge” technique to treat PCLA, “suture bridge” fixation can provide sufficient limiting load and stress on an avulsed fracture block to maximize the surface area of bone healing.^7^ Moreover, the application of the "suture bridge" fixation allows the bone to be securely attached to the surface and reducing the risk of avulsion.^8^

Our surgical technique uses FiberWire for "suture bridge" fixation. A study on the treatment of PCLA with arthroscopic FiberWire fixation suggests that FiberWire has greater mechanical strength and flexibility than conventional sutures. Moreover, when the fracture block is fixed by FiberWire, the fracture block is allowed to move slightly, which conforms to the principle of biomechanical fixation.^9^

When FiberWire is wrapping the PCL during surgery, the separation of the PCL from the plate-femoral ligament must be gently conducted. If the 2 are intertwined together, the loose wrapping of the FiberWire can cause its fixed position to be breached. During this process, the package should not be excessively tightened to avoid damage to the PCL. The distance between the 2 tibial tunnels should be appropriate so that the 2 FiberWires form a "W"-shaped structure behind the PCL. This step will ensure that the pressure is evenly distributed to the maximum extent and the fixation of the fracture block is more stable. Table 1 lists the advantages and disadvantages of this technique, and Table 2 includes pearls and pitfalls. The arthroscopic "suture bridge" double posterior internal portal to fix posterior cruciate ligament tibial avulsion is a reliable surgical treatment.

**Table 1:** Advantages and Disadvantages

Advantages
The "suture bridge" technique of fixing PCLA can ensure the even distribution of the suture and reduce the cutting of PCL parenchymal fibers.
The surgery does not use metal implants and does not require a revision surgery to remove them, thereby reducing the financial burden on patients and the risk of a second surgery.
Compared with the combination of the posterior and posterolateral portals, the establishment of the dual posterior internal portal preserves the integrity of the posterior septum and reduces the loss of blood flow caused by the destruction of the posterior septum, thereby facilitating the healing of fracture block.
Disadvantages
The establishment of a double posteromedial portal requires a long learning time and may damage the saphenous nerve and great saphenous vein in the process.
There is a risk of fixation failure in patients with osteoporosis when the distal tibial FiberWire is fixed with an outer-row anchor.
Compared with open internal fixation operation, "suture bridge" still runs the risk of fixation and displacement of the fracture block.

PCL, posterior cruciate ligament; PCLA, posterior cruciate ligament avulsion.

**Table 2:** Pearls and Pitfalls

Pearl:
Double posteromedial portal can reduce the risk of intraoperative damage to the posterior septum.
The “W” shaped structure formed by the two Fiber Wires allows for better fixation of the fracture.
Pitfalls:
When applying the FiberWire to wrap the PCL, the ligation of other ligaments (such as the meniscofemoral ligament and the anterior cruciate ligament) should be prevented to avoid unstable fixation of the tail line of the distal tibia suture.
An outer-row anchor should be implanted to fix the distal tibial Fiber Wire under 90° knee flexion and anterior drawer test to restore PCL tension.

PCL, posterior cruciate ligament.

## Disclosures

J.D. reports financial support was provided by National Natural Science Foundation of China, Hebei Provincial Department of Science and Technology, and Hebei Medical University. All other authors (S.W., R.Z., Y,Z., Y.L., Y.L., X.M.) declare that they have no known competing financial interests or personal relationships that could have appeared to influence the work reported in this paper.

## Supplementary Data

Video 1 After preoperative preparation, The anteromedial portal and anterolateral portal were created. The tissue between the ACL and PCL was cleaned off by a shaver. The pathway between the PCL and the medial wall of intercondylar notch was opened. Then the double posteromedial portals were created, the LPM was used as observing portal and HPM was used as working portal. The first FiberWire was put into joint cavity from anterolateral portal. One end was passed through the pathway between the PCL and the medial wall of the intercondylar notch , the other end through the ACL and PCL pathway. Then a 2.5-mm guide needle was used to drill from the anterior to the posterior. The second fiber wire was put from anterolateral portal and repeat tunnel 1 procedure. Repeat tunnel 1 procedure. Drill out tunnel 2 and Locating medial and inferior of the fracture bed. Adjust the “suture bridge.” At last the stability of the knee was checked by a posterior draw test. (ACL, anterior cruciate ligament; HPM, high posteromedial; LPM, low posteromedial; PCL, posterior cruciate ligament.)
